# Autism Spectrum Disorder Associated With Gut Microbiota at Immune, Metabolomic, and Neuroactive Level

**DOI:** 10.3389/fnins.2020.578666

**Published:** 2020-10-08

**Authors:** Enriqueta Garcia-Gutierrez, Arjan Narbad, Juan Miguel Rodríguez

**Affiliations:** ^1^Gut Microbes and Health Institute Strategic Program, Quadram Institute Bioscience, Norwich, United Kingdom; ^2^Department of Nutrition and Food Science, Complutense University of Madrid, Madrid, Spain

**Keywords:** autism spectrum disorder, gut microbiome, gut-brain axis, biomarker, neurotransmitter, GABA, serotonin

## Abstract

There is increasing evidence suggesting a link between the autism spectrum disorder (ASD) and the gastrointestinal (GI) microbiome. Experimental and clinical studies have shown that patients diagnosed with ASD display alterations of the gut microbiota. These alterations do not only extend to the gut microbiota composition but also to the metabolites they produce, as a result of its connections with diet and the bidirectional interaction with the host. Thus, production of metabolites and neurotransmitters stimulate the immune system and influence the central nervous system (CNS) by stimulation of the vagal nerve, as an example of the gut-brain axis pathway. In this review we compose an overview of the interconnectivity of the different GI-related elements that have been associated with the development and severity of the ASD in patients and animal models. We review potential biomarkers to be used in future studies to unlock further connections and interventions in the treatment of ASD.

## Introduction

**FIGURE 1:**
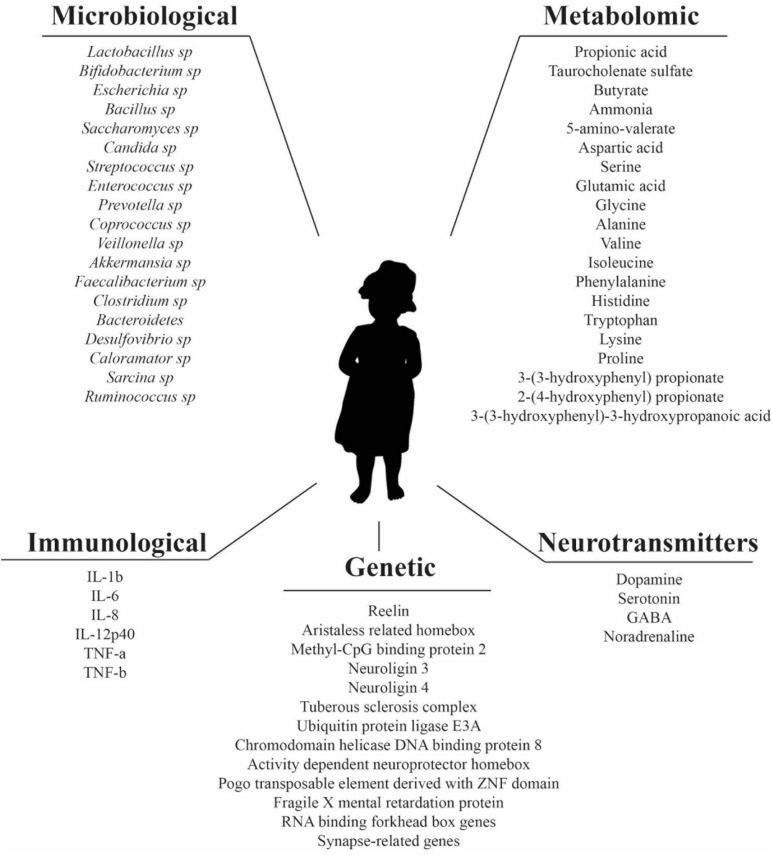
Microbiological, metabolomic, immunological, genetic factors, and neurotransmitters reported in literature as altered in ASD patients.

Autism spectrum disorder (ASD) is a group of brain developmental disorders characterized by stereotyped behavior and deficits in communication and social interaction. Initially, it was believed that ASD had an environmental origin. However, at the moment it is accepted that ASD development is the result of multiple factors, including environmental, genetics, and neurodevelopmental (Rylaarsdam and Guemez-Gamboa, 2019). The prevalence of ASD in the development of children and on society constitutes an economic burden for families, where the main costs are associated to special education and the loss of productivity of the parents (Buescher et al., 2014; Christensen et al., 2018). Additionally, it has been reported that over the last decades, there is an increasing prevalence of ASD, reaching 1 in 132 globally (Matson and Kozlowski, 2011; Baxter et al., 2015; Hansen et al., 2015). Therefore, there is a need to develop and implement effective interventions. However, there is no defined etiology and pathology for ASD, and this limits the development of specific therapies (Rossignol and Frye, 2012). Previous studies have shown that there are several factors that might have an influence on development and prognosis of ASD, such as genetics, immunological, inflammatory, environmental, and more recently, the gut microbiota (Fakhoury, 2015). Genetic factors thought to be involved in processes such as synapse formation, transcriptional regulation or pathways for chromatin-remodeling are listed in Figure 1 (Rylaarsdam and Guemez-Gamboa, 2019). However, genetic factors in ASD development are not the focus of this review and this subject is reviewed elsewhere (Chaste and Leboyer, 2012; Huguet and Bourgeron, 2016; Rylaarsdam and Guemez-Gamboa, 2019).

The gut harbors millions of microorganisms linked by complex ecological relationships between them and the host, often mediated by the production of metabolites. The gut microbiota has been proposed as a key element involved in many conditions, such as obesity, colorectal cancer, irritable bowel syndrome (IBS), diabetes type 2, rheumatoid arthritis, Parkinson’s disease, and Alzheimer’s disease and also with cognitive conditions such as anxiety, depression, and autism (Ceppa et al., 2019). The gut-brain axis theory, now well-established and accepted, states that the gut and the brain communicate and influence each other (Bienenstock et al., 2015; Mayer et al., 2015; Cryan et al., 2019). The gut-brain axis theory has its origin on the observation of the improvement of patients diagnosed with hepatic encephalopathy, after antibiotic treatment (Carabotti et al., 2015). Moreover, IBS and its gut microbiota alterations have been linked to anxiety and depression (Simpson et al., 2020). There is even recent evidence suggesting that human personality traits might be associated with the gut microbiome (Johnson, 2020).

Increasing evidence shows that gastrointestinal (GI) symptoms, such as gastrointestinal disruption, abdominal pain, diarrhea, constipation, and flatulence, has been characterized as a common comorbidity in patients with ASD, ranging between 9 and 84% depending on the studies being retrospective or prospective (Wasilewska and Klukowski, 2015), and are linked to the severity of ASD symptoms (Adams et al., 2011; Gorrindo et al., 2012; Chaidez et al., 2014). However, cause-effect relationship between GI symptoms and ASD has not been proven yet. In fact, it has been suggested that GI symptoms should be considered as part of the ASD phenotype, like the behavioral symptoms (Niesler and Rappold, 2020). On the other hand, there are studies that have demonstrated that the administration of a single strain, such as human commensal *Bacteroides fragilis*, is able to ameliorate social deficit in a mice model (Hsiao et al., 2013). Moreover, *B. fragilis* corrected gut permeability and altered microbial composition. Additionally, treatments such as Microbiota Transfer Therapy (MTT), focused on gut microbiota regulation, have shown promising results by improving ASD-related symptoms in patients that were sustained after finishing the treatment (Kang et al., 2019). These improvements were reported hand-in-hand with an increment in bacterial diversity and relative abundances of *Bifidobacterium* and *Prevotella*. Overall, these evidences suggest a potential correlation between these factors and communicative defects and stereotypic behavior associated to ASD that needs to be further explored. The validation of biomarkers related to the gut-brain axis would be of great value in the diagnosis, the development, and the follow-up of potential therapies for patients with ASD. This review will focus on the role of the gut microbiota in the pathology of ASD via the gut-brain axis and the related biomarkers that have been described in the literature.

## The Microbiota in the Gut

It has been reported that the human gut carries more bacterial cells than human cells are in the entire body, and that the metagenome of the gut microbiota encodes approximately eight million genes, in contrast to the approximately 23,000 genes encoded in the human genome (Ceppa et al., 2019). The gut ecosystem comprises the bacteria, archaea, viruses, fungi, yeast, and eukarya (Ceppa et al., 2019). Gut microbiota is not uniformly distributed across the GI tract. The distribution depends on the combination of factors such as pH, water activity or gas composition that fluctuate through the gut and have been reviewed previously (Lozupone et al., 2012; Donaldson et al., 2016; Garcia-Gutierrez et al., 2018). The gut microbiota composition also varies over life span. At the moment, there is some controversy on how sterile the placenta is and whether the meconium of healthy new-borns contain traces of microbiota (DiGiulio et al., 2008; Jiménez et al., 2008; Moles et al., 2013; Aagaard et al., 2014; Rodríguez et al., 2015; de Goffau et al., 2019). However, the process of a succession of bacterial colonization events in the gut begins at birth, via the microbiome of the maternal vagina during the delivery (or the skin in the case of a C-section), and introduction of new species in the human gut through feeding (human milk first and solid food after weaning), after the delivery (Dominguez-Bello et al., 2010; Fernandez et al., 2013; Bokulich et al., 2016; Yassour et al., 2016). This changing composition stabilizes after the third year of life and it is maintained during the adult life. There is evidence of gut microbiota changes during senescence and these changes might be related to the conditions developed over this period, such as cognitive impairment or elderly malnutrition (Nagpal et al., 2018; Xu C. et al., 2019). As a general feature, gut microbiota is usually resilient and recovers after acute changes, like consumption of antibiotics. However, structural composition of the gut microbiota is determined by sustained factors like lifestyle or diet (Conlon and Bird, 2015; Singh et al., 2017; Ceppa et al., 2019).

There are five major bacterial phyla in the gut, Bacteroidetes, Firmicutes, Actinobacteria, Proteobacteria, and Verrucomicrobia (Donaldson et al., 2016). The different conditions, such as presence of bile acids, oxygen, or nutrient availability, across the GI tract result in different distribution of these groups. Thus, the small intestine is colonized by representatives of groups of facultative anaerobes of Firmicutes (lactobacilli) and Proteobacteria (enterobacteria), while the colon is colonized mainly by fermentative organisms from *Bacteroidaceae*, *Prevotellaceae*, and *Rikenellaceae* families (Bacteroidetes) and *Lachnospiraceae* and *Ruminococcaceae* families (Firmicutes) (Donaldson et al., 2016). The characterization of the gut microbiota has been mainly conducted by analyzing fecal samples, however, this might provide a false image of the proportion and diversity of the gut microbiota composition (Rodríguez et al., 2015). Regardless of the potential artifact of the compositional information, functionality is the key factor for a balanced microbiota. Despite that it is not possible to define a healthy gut microbiota in terms of taxonomical composition, it has been suggested that metabolic functionality of pathways remains redundant in the gut microbiome, and it is the source of a balanced equilibrium and resilience after acute perturbances (Lozupone et al., 2012). The identification of biomarkers as gene functions associated to a balanced gut health and specific pathologies will favor and improve the development of efficient microbiota-associated treatments in the future.

## The Microbiota-Gut-Brain Axis

The gut–brain axis is considered a bidirectional pathway for the communication between the gut and the brain. However, this concept can be expanded to include also the microbiota as a key element in this triangle (Cryan et al., 2019). The importance of the microbiota in this relationship has been established via different routes. Studies conducted in germ-free animals have provided evidence that the brain was affected when the gut microbiota was not present (Diaz Heijtz et al., 2011; Cryan et al., 2019). Other studies have shown that alterations in behavior in animals were induced by providing specific strains of bacteria, and those observations were sustained in human studies afterwards, e.g., *Bifidobacterium longum* strains 1714 and NCC3001 (Allen et al., 2016; Pinto-Sanchez et al., 2017). Exposition to infections showed alteration in gut-brain symptoms, and immune activation, and the use of antibiotics affected the central nervous system (CNS) and the enteric nervous system (ENS). In the reverse situation, hepatic encephalopathy has been successfully treated with microbiota-targeting antibiotics (Collins, 2016). Extensive work with mice models has shown that there are several processes in the nervous system that are linked to the regulatory effect of the gut microbiota, including neurogenesis in hippocampus, the amygdala, myelination, the length, and spine density, the synaptic connections, the microglia and the permeability of the blood-brain-barrier (BBB) (Cryan et al., 2019). Another process where microbiota is involved is in the synaptic and neuronal plasticity. Studies with germ-free mice indicated low levels of expression of brain-derived neurotrophic factor (BDNF) in the cortex and hippocampus. BDNF is associated with brain plasticity and has a regulatory function on neural growth (Leung and Thuret, 2015). BDNF is involved in many learning and behavioral processes, especially the ones associated with hippocampal learning and working memory (Gareau et al., 2011). The receptors for N-methyl-D-aspartate (NMDAR) are also closely involved in the synaptic plasticity and cognitive function and the production of NMDAR is connected to the levels of BDNF (Maqsood and Stone, 2016). Low levels of BDNF in germ-free mice successively lowers the NMDAR production, which also affects γ-aminobutyric acid (GABA) inhibitory interneurons and ultimately this translates into cognitive deficits (Maqsood and Stone, 2016). Gut microbiota exerts its action over BDNF by alterations in neurotransmitter and modulatory pathways, such as the kynurenine, involved in tryptophan metabolism and by the action of the short chain fatty acids (SCFAs) (Cryan et al., 2019). Studies in mice have shown that depleted BDNF levels could be recovered by the direct administration of a strain of *B. longum* subsp. *infantis* (Bercik et al., 2012) and other probiotics, prebiotics, and antimicrobials that increase the proportion of lactobacilli, Firmicutes, and Actinobacteria and decrease the Proteobacteria and Bacteroidetes levels in the gut, suggesting potential interventions to target key regulatory elements in the CNS (Maqsood and Stone, 2016).

The microbiota in the gut and the brain can communicate through a variety of routes that involve neuroendocrine, neuroimmune, and autonomic nervous systems pathways (Grenham et al., 2011; Mayer, 2011). The immune system is particularly important (Carlessi et al., 2019) where cytokines components of the immune system communicate directly with the brain via the vagal nerve inducing changes in the BBB (Quan, 2008). It also affects the hypothalamic pituitary adrenal (HPA) axis, that centralizes the stress response system, stimulated by physical or psychological situations (Scriven et al., 2018). HPA axis alterations have been reported in post-traumatic stress or depression. A number of bacterial strains (e.g., *Lactobacillus farmicinis*) can modify such changes via impacting the gut permeability or balancing levels of adrenocorticotropic hormone (ACTH), corticosterone and BDNF (*Bifidobacterium infantis*) (Desbonnet et al., 2008; Ait-Belgnaoui et al., 2012). The vagus nerve is responsible for many anti-inflammatory effects through the contact with the HPA axis and other pathways, such as the cholinergic anti-inflammatory and the splenic sympathetic anti-inflammatory (Forsythe et al., 2014). The vagus nerve interacts with bacteria via SCFAs that cross the gut wall, and it can even differentiate between pathogenic and non-pathogenic bacteria (Bonaz et al., 2018). The function of many probiotics is also determined by their interactions with the vagus nerve. Bacteria can also produce and secrete neurotransmitters. Some representatives of the genera *Lactobacillus* and *Bifidobacterium* can produce GABA, while representatives of *Escherichia*, *Bacillus*, and *Saccharomyces* can produce noradrenaline (Barrett et al., 2012). Serotonin (5-hydroxytryptamine, 5-HT) is a product of some species of *Candida*, *Streptococcus*, *Escherichia*, and *Enterococcus* and it is mediated by tryptophan (Scriven et al., 2018). Other neurotransmitters produced by bacteria are dopamine (*Bacillus*) and acetylcholine (*Lactobacillus*) (Dinan et al., 2015). Gut microbiota is the key element that controls tryptophan catabolism via the kynurenine pathway, the primary pathway for tryptophan catabolism (Ceppa et al., 2019). Changes in the serotonergic system have been associated with depression and IBS (Owens and Nemeroff, 1994). Gut microbiota also produce metabolites as a result of the fermentative process during the food digestion in the gut. Metabolites are the result of the breakdown of carbohydrates, polyphenols, lipids, and proteins, together with gasses (carbon dioxide, hydrogen, and methane) and the production of energy. The diet composition will result in different types of SCFA that communicate with the brain through the vagus nerve, producing different effects on the nervous system (Silva et al., 2020). Butyric acid has been associated with satiety, and high levels of propionic acid (PAA) have been linked with ASD (Shultz and MacFabe, 2014; Abdelli et al., 2019). The role of PAA is particularly interesting after the observations that ASD behavioral effects in children worsen after the consumption of high levels of PPA (Meeking et al., 2020). Moreover, supplementation with PPA in animal models led to behavioral effects similar to ASD while metabolic impairment of glutathione, carnitine, and fatty acids (FA) has been observed in the serum of ASD patients receiving PPA (Frye et al., 2013). PPA can accumulate in the cells and alter neuronal communication by its impact on neurotransmitter release, gap junctions and intracellular calcium release, among other effects that will be discussed later. Overall, these studies also suggest that dietary components can play a major role in the selection of bacteria and the production of metabolites, that ultimately will affect the gut-brain axis.

Despite that many studies have established these pivotal connections between the gut and the brain, translational human studies are particularly needed to understand the mechanisms underlying the microbiota-gut-brain axis. This will be key to design microbial-based interventions and therapeutic strategies to target neuropsychiatric disorders.

## Potential Relationships Between the Microbiota and ASD

There are a variety of factors that seem to connect gut microbiota with ASD symptoms. Early life events, such as delivery mode, have a huge impact on the composition of the microbial communities. Infants delivered by C-section showed a higher probability of developing ASD in comparison to the children delivered vaginally (odds ratio of 1.23) (Curran et al., 2015). Other prenatal factors, such as gestational diabetes or maternal obesity during pregnancy, can modify the gut microbiota (Connolly et al., 2016). In a mice model, when mothers where fed a high-fat diet, it induced dysbiosis and autism-like phenotypes (Buffington et al., 2016). Additionally, in children with diagnosed ASD an increased use of antibiotics was reported in comparison with controls (Atladottir et al., 2012). An explanation might be the effect of antibiotics on the gut microbiota (Bokulich et al., 2016). The effect of antibiotics on the gut microbiota has been extensively studied in recent times. Antibiotics not only target pathogens, they also affect commensal bacteria that contribute to the gut homeostasis (Mu and Zhu, 2019; Sun et al., 2019). Sometimes, the impairment produced by the use of antibiotics, depending on factors such as the type of antibiotic, length of treatment or age of the host, can be overcome and the balanced restored in the gut microbiome communities (Langdon et al., 2016). However, in other cases, the use of antibiotics leads to the loss of key species in the microbiome, producing lifelong phenotype alterations, such as obesity (Wang et al., 2017). The effects of antibiotics on the gut microbiota of children can be more detrimental. Thus, the composition of the microbiota of children of less than 3 years who were treated with antibiotics was less diverse (Yassour et al., 2016). Even the antibiotics used during pregnancy seem to be correlated with a higher risk factor for the development of ASD (Atladottir et al., 2010). Another important factor is the early feeding pattern. Infants fed with formula milk showed higher levels of *Clostridium difficile* in comparison with infants who were breast fed (Azad et al., 2013). Additionally, breastfeeding over 6 months has been associated with a lower risk of ASD development (Schultz et al., 2006) and ASD-related GI symptoms (Penn et al., 2016).

GI symptoms are a comorbidity reported in 9–84% of ASD patients (Wasilewska and Klukowski, 2015). These include constipation (20%) and diarrhea (19%), which is more frequent in children with ASD than in their unaffected brothers or sisters (42 vs. 23%, respectively) (Wang et al., 2011). The evidence linking directly or indirectly the gut microbiota with ASD symptoms shows that this might happen partially by its influence on the host metabolism and the immune system (de Angelis et al., 2015; Mead and Ashwood, 2015).

The “leaky gut” or increased permeability of the intestinal epithelium, is one of the conditions reported in ASD patients (Quigley, 2016), where 36.7% of ASD patients and their relatives (21.2%) showed higher percentage of abnormal intestinal permeability in comparison to the control group (4.8%) (de Magistris et al., 2010). As a result of increased permeability, toxins and bacterial products can get into the bloodstream, ultimately affecting brain function and impairing social behavioral scores (Emanuele et al., 2010; Onore et al., 2012; Hsiao et al., 2013). There are a few elements that are used to measure the integrity of both the gut barrier and the BBB, like claudin (CLDN)-5, CLDN-12, CLDN-3, and MMP-9, increased in the ASD-patients’ brain, and the intestinal tight junction components (CLDN-1, OCLN, TRIC), decreased in ASD patients (Fiorentino et al., 2016). The lactulose: mannitol test is used to measure intestinal permeability, and it is increased in ASD patients when compared with healthy controls (de Magistris et al., 2010). On the other hand, bacterial products such as acetate and propionate may enhance the integrity of the BBB (Braniste et al., 2014). The leaky gut also increases the antigenic load from the gastrointestinal tract. Thus, lymphocytes and ASD-associated cytokines, like interleukin-1β (IL-1β), IL-6, interferon-γ (IFN-γ), and tumor necrosis factor-α (TNF-α), circulate and cross the BBB. IL-1β and TNF-α are responsible for inducing immune responses in the brain by binding to the brain endothelial cells (de Theije et al., 2011).

One of the common changes observed in ASD patients and animal models relates to the composition of the gut microbiota and their metabolic products (de Magistris et al., 2010; Borre et al., 2014; Kushak et al., 2016). It was found that the gut microbiota of children with ASD was less diverse and exhibited lower levels of *Bifidobacterium* and Firmicutes and higher levels of Bacteroidetes, *Lactobacillus, Clostridium, Desulfovibrio, Caloramator*, and *Sarcina*, than that of children without ASD (de Angelis et al., 2013). ASD children with GI symptoms had lower abundances of *Prevotella, Coprococcus*, and unclassified *Veillonellaceae*, than symptom-free neurotypical children (Kang et al., 2013). A recent systematic review and meta-analysis identified approximately 431 studies conducted in ASD patients that involved analysis of the gut microbiota, although many of these studies did not provide quantitative data (Xu M. et al., 2019). The meta-analysis revealed significant differences between gut bacterial groups. Thus, ASD patients had a lower percentage of *Akkermansia* and *Bacteroides* when compared to controls. *Bacteroides* are known for inducing anti-inflammatory effects (Bolte, 1998). Another important group traditionally associated with beneficial effects in the human gut is *Bifidobacterium*, with significantly lower abundance in ASD patients (Xu M. et al., 2019). On the other hand, the analysis of five studies showed that the percentage of sequences of the genus *Faecalibacterium* was significantly higher among ASD patients (Finegold et al., 2010; de Angelis et al., 2013; Kang et al., 2013; Inoue et al., 2016; Strati et al., 2017; Xu M. et al., 2019). Higher relative abundance of lactobacilli (generally considered to be beneficial bacteria) has been observed in children diagnosed with ASD although it may reflect an effect of the diet (e.g., a high consumption of yogurt and yogurt-like fermented milks). Several studies highlighted the relevance of other bacterial groups, like the *Clostridium histolyticum* group (*Clostridium* clusters II and I), which were present in higher levels in fecal samples of ASD children (Parracho et al., 2005). *Clostridium* is known for producing neurotoxins that might have systemic effects (Parracho et al., 2005). It was observed that reductions of this *Clostridium* group brought significant improvements in children with ASD-like symptoms (Sandler et al., 2000). *Ruminococcus* is another genus associated with ASD symptoms and functional GI disorder (Joossens et al., 2011; Xu M. et al., 2019).

Despite many different studies demonstrate alterations of the gut microbiota in ASD patients, others have not described this association. To illustrate this, a study comprising 59 ASD individuals and 44 normal siblings found no significant difference between them in relative abundances of total Bacteroidetes, *Sutterella* or *Prevotella* (Son et al., 2015). Additionally, there is a lack of studies that evaluate the role of gut mycobiome and gut virome in ASD. Increasing evidence suggests that mycobiome might be a key element in maintaining a gut-brain axis balanced dynamics due to its close interaction with the gut bacteria (Huseyin et al., 2017a,b). An increased abundance of *Candida* in the gut mycobiome composition of ASD patients was reported for the first time recently (Strati et al., 2017; Enaud et al., 2018). It was hypothesized that its interaction with other microbes, such as lactobacilli, might have an effect on the immune system via pro-inflammatory effectors and prevent the recovery of the balanced gut microbiota (Enaud et al., 2018). In any case, the interactions between bacteria and other members of the gut microbiota could bring valuable information about their role in ASD condition.

## Gut Microbiota-Mediated Metabolites as Biomarkers

Gut microbiota products include a variety of metabolites, such as SCFAs, phenolic compounds and free amino acids (FAA), that affect the behavior of ASD patients. It is believed that this effect is mediated via the vagal pathways (Macfabe, 2012).

Short chain fatty acids include acetic acid (AA), PPA, butyrate, isobutyric acid, valeric acid, and isovaleric acid, as products of the fermentation of non-digestible carbohydrates by gut bacteria (Al-Lahham et al., 2010). SCFAs have beneficial effects on the human host, like improvement in glucose metabolism, energy homeostasis, reductions in body weight and the risk of colon cancer (Chambers et al., 2015). PPA is produced mainly by Bacteroidetes, *Clostridium*, and *Desulfovibrio* and can cross the BBB. As stated before, PPA can inhibit the Na+/K+ ATPase, increase NMDA receptor sensitivity and alter mitochondrial and fatty acid metabolism. It also can trigger immune activation and changes in gene expression (Meeking et al., 2020). PPA has been linked to the development of ASD-like behaviors (MacFabe et al., 2007, 2011; Shultz et al., 2008; Ossenkopp et al., 2012). In a mice model, the administration of high doses of PPA induced some autistic-like behaviors (Thomas et al., 2012). In a rat model, the intraventricular administration of PPA induced hyperactivity and repetitive behaviors in a similar way to the behavioral changes in ASD patients (MacFabe et al., 2007). Additionally PPA led to impaired social behavior in rats, probably due to the alteration of dopamine and serotonin levels (Mitsui et al., 2005). Butyrate has shown anti-inflammatory effects and ability to modulate the synthesis of dopamine, norepinephrine, and epinephrine (Gualdi et al., 2008; Cleophas et al., 2016).

Free amino acids are derived from the hydrolysis of the proteins and peptides and their fecal levels were higher in ASD children with symptoms in comparison with healthy children and, more specifically, the levels of the amino acids Asp, Ser, Glu, Gly, Ala, Val, Ile, Phe, His, Tpr, Lys, and Pro (de Angelis et al., 2015). The levels of some of them, particularly Glu, a neurotransmitter in the CNS, is altered in other neuropsychiatric disorders (Sheldon and Robinson, 2007; Shimmura et al., 2011). Tryptophan, the precursor of GABA was increased in the urine of ASD patients and tryptophan fragments were also found in the urine of patients with depression and intellectual disability (Noto et al., 2014). Other compounds found in higher levels in the urine of ASD children were 2-(4-hydroxyphenyl) propionate and taurocholenate sulfate, while 3-(3-hydroxyphenyl) propionate and 5-amino-valerate were found in lower levels (Ming et al., 2012). A phenylalanine metabolite [3-(3-hydroxyphenyl)-3-hydroxypropanoic acid], produced by *Clostridia* spp., was increased in the urine of ASD patients and was linked to ASD-like behaviors in mice models (Shaw, 2010).

## Biomarkers From the Immune System Pathways

The gut and the brain can also influence each other via immunological pathways, and microbial diversity is key to maintaining immune homeostasis, as it is linked to the development of the gut-associated lymphoid tissue (GALT) (Rodríguez et al., 2015; Ceppa et al., 2019). The GALT recognizes pathogenic microorganisms and mediates a defense response. GALT is known for producing IgA, modulating innate immune responses when bacterial cells come in contact with dendrites of the ENS (Ceppa et al., 2019). IgAs also recognizes and binds to specific undesired microorganisms to facilitate their removal in feces, while maintaining the commensal bacteria (Lebeer et al., 2010). When there is impairment of the gut microbiota balance, one of main effects is the development of inflammatory processes. A correlation has been established between inflammation and immune dysfunction in ASD patients (Fattorusso et al., 2019). In fact, a comparison between transcriptomics profiles on ileal and colonic tissues showed similarities between ASD and inflammatory bowel disease (IBD) patients (Fattorusso et al., 2019).

There are different inflammatory markers for ASD that have been described in literature, but with limited consensus. For example, IgA has been found to increase in ASD patients in some studies, but not in others (Kushak et al., 2016). Levels of pro-inflammatory cytokines, such as IL-1β, IL-6, IL-8, and IL-12p40, have been shown to be increased in the plasma of ASD patients (Ashwood et al., 2011). Also, TNF-α and transforming growth factor (TGF-β) have been linked to the severity of the ASD symptoms. Some probiotics, including strains belonging to the species *Lactobacillus sakei*, *Lactobacillus reuteri*, *Lactobacillus paracasei*, *Lactobacillus plantarum*, *Lactobacillus acidophilus*, *Lactobacillus salivarius* and *Bifidobacterium breve*, modulate or inhibit the production of pro-inflammatory cytokines IL-8, TNF-α, and IFN-γ and increase the anti-inflammatory cytokine IL-10 (Thomas and Versalovic, 2010; Ganguli et al., 2013).

The toxins produced by the pathogenic members of the microbiota increase gut permeability, developing impaired intestinal barrier and allowing the translocation of the gut bacteria through the intestinal wall into the mesenteric lymphoid tissue, inducing the activation of the immune system (Dicksved et al., 2012). This, in turn, releases the inflammatory cytokines and activates the vagal system, regulating CNS activity (Yarandi et al., 2016). The peripheral cytokines are able to induce a behavior linked to depression via the vagal nerve (Konsman et al., 2000). Also, other metabolic compounds produced by gut microbiota, such as lipopolysaccharide (LPS), enter the blood through the impaired gut wall and activate Toll-like receptors in the ENS and CNS (Abreu, 2010). The immune response in mediated by IgE in the gut, where it raises serotonin levels and reduces 5-hydroxyindoleacetic acid (5-HIAA) ones in the gut, which has been linked to reduced social communication and increased repetitive behavior (Li et al., 2017). Additionally, an activation of the neuroendocrine system and downregulation of the dopamine activity in the prefrontal cortex were also observed in a mice model (de Theije et al., 2014). ASD patients also have higher levels of zonulin in plasma, a protein that modulates gut permeability, and its levels seem to be associated with the severity of the ASD symptoms (Fattorusso et al., 2019).

The immune system is, therefore, closely linked to the effect of microbiota on the gut epithelial permeability connecting the gut and brain through neuroendocrine and neuroimmune pathways that ultimately modulates ASD severity.

## Neuroactive Compounds as Biomarkers

Sensory hyper- and hypo-responsiveness are typically characteristic of autistic patients even though they are not part of the core definition of ASD. However, diet and probiotic interventions might alleviate them. A variety of neuroactive compounds that activate or inhibit central neurons are produced by gut microbiota, such as serotonin, GABA, dopamine (DA) and histamine (Eisenstein, 2016; Spiller and Major, 2016).

The first identified ASD biomarker was serotonin, proposed as a link for the gut-brain axis (Mulder et al., 2004). Serotonin is synthesized in the intestines and the brain, and is thought to be involved in the development of the CNS and the ENS (Gaspar et al., 2003). Children with ASD showed higher levels of serotonin in blood that is believed to be caused by its gastrointestinal hypersecretion (Israelyan and Margolis, 2019). It was believed that genetic factors might be the cause of this overproduction. It is believed that infections, gastrointestinal disorders, such as gut microbiome dysbiosis, and immune system impairment might also be involved in higher levels of serotonin in ASD patients (Fattorusso et al., 2019). A higher prevalence of clostridia in the gut mucosa of children with ASD and GI disorders was associated with higher levels of cytokines, serotonin, and tryptophan in biopsies (Luna et al., 2017). In addition, higher levels of tryptophan (the precursor of serotonin) in the GI tract of ASD children were associated with more severe ASD behavioral symptoms, and with a lower availability and synthesis of serotonin in the brain (Luna et al., 2017). Therefore, gut microbiota dysbiosis affects the availability of tryptophan for the host and worsens cognitive impairment. Interventions to regulate gut dysbiosis might improve the ASD symptoms. It has been observed that, in a mice model, the offspring’s brains from mice exposed to valproic acid (VPA) showed ASD behavior alterations in the microbiota and lower levels of serotonin (de Theije et al., 2014). However, other strategies, such as addition of tryptophan to the diet and administration of serotonin reuptake inhibitor have not improved ASD behaviors (Muller et al., 2016).

Gamma-aminobutyric acid is the main inhibitory neurotransmitter in the brain. It has been observed that an altered GABA pattern is a key characteristic of the neurophysiology of ASD. A recent study was performed on the effect of GABA in the brain regions that are critical to sensory functions and higher-order motor, including the primary visual cortex, the left supplementary motor area (SMA), the left sensorimotor cortex, and the left ventral premotor cortex (vPMC) (Umesawa et al., 2020). Sensory processing is considered abnormal in autism at input, cognitive and behavioral reactivity levels, potentially involving processes of high cognitive processing (Thye et al., 2018). If the inhibitory GABAergic transmission is impaired in ASD patients, it may result in an abnormal balance of excitation/inhibition in the brain, alteration of neural signaling, processing of information and responding behavior (Foss-Feig et al., 2017). The reduced levels of GABA in the higher-order motor areas, integrating multiple sensory modalities, might be behind the sensory hyper-responsiveness in ASD patients (Umesawa et al., 2020). This correlates with a previous study reporting that GABA levels in processing touch areas were related to tactile hypersensitivity, frequently observed in in ASD patients (Sapey-Triomphe et al., 2019). Moreover, when mice where administered with *Lactobacillus rhamnosus* JB-1, there was a stimulation of the transcription of GABA receptors in the vagus nerve, which induced behavioral and psychological effects that were reverted after vagotomy (Bravo et al., 2011).

Other studies in animal models have shown that impaired learning and increased depression-like behaviors were observed in a mice model, after the depletion of the gut microbiota by antibiotics. This was correlated with alterations in the levels of 5-hydroxyindoleacetic acid, 5-HT, homovanillic acid, DA, and noradrenaline, and in the mRNA levels of the corticotrophin-releasing hormone receptor 1 and the glucocorticoid receptor (Hoban et al., 2016). Another type of intervention involves using epigenetic dysregulation as a pharmacological target. Thus, it was shown that sodium butyrate, acting as a histone deacetylase inhibitor, improved social and repetitive behavior in BTBRT +tf/J (BTBR) mice. The administration of sodium butyrate had an effect on the transcriptome of several neurotransmitters and regulatory genes (Kratsman et al., 2016). Overall, these studies reinforce the correlation between ASD symptoms, gut microbiota and levels of neurotransmitters, and suggest that interventions focused on neurotransmitters could have the potential to reduce ASD behavioral symptoms.

## Use of the Gut Microbiota Modulation as a Potential Therapy for ASD Patients

At the moment, there are no effective therapies for treating ASD patients. In fact, research on autism is currently focusing on strategies for alleviating symptoms of ASD patients rather that looking for a cure (Willingham, 2020). Modulation of the gut microbiota has arisen as a potential therapy through interventions using probiotics, prebiotics, fecal microbiota transplantation (FMT) and diet.

### Probiotic Interventions

The use of probiotics have displayed promising results in prevention and treatment of conditions such as obesity, colorectal cancer, IBD, IBS, or depression in human studies and animal models (Walsh et al., 2014; Sharma and Shukla, 2016; Valsecchi et al., 2016). One of the investigated areas is the prevention of inflammation by regulating the barrier function, including the expression of tight junction proteins. Some studies have shown alleviation of GI symptoms and immunomodulation of cytokines using *B. longum* subsp. *infantis* 35624 (O’Mahony et al., 2005; Whorwell et al., 2006), *Lactobacillus helveticus* R0052 and *Bifidobacterium longum* R0175 (Messaoudi et al., 2011), *Lactobacillus casei* Shirota (Rao et al., 2009), *L. plantarum* WCFS1 (Karczewski et al., 2010) or *Lactobacillus rhamnosus* GG (Patel et al., 2012).

*Bacteroides fragilis* was used in a treatment that reduced ASD-like behavior in a rodent model of ASD (Hsiao et al., 2013). This bacterium reduced gut permeability and modulated the gut microbiota composition, suggesting that the key factors for the treatment of relieving ASD-like behaviors in patients should aim to balance gut microbiota and enhance the gut barrier. Another study used oral supplementation with a *L. acidophilus* strain and reported reduced levels of D-arabinitol in the urine of ASD children, improving the ability to follow directions (Kaluzna-Czaplinska and Blaszczyk, 2012). In a case study that used VSL#3, a mixture of 10 probiotic strains, it was reported to relieve and improve GI symptoms and other characteristics of ASD (Grossi et al., 2016). However, despite the overall positive outcome of probiotics in the treatment of symptoms of ASD patients, large randomized controlled studies are missing.

### Prebiotic Interventions

Prebiotics are non-digestible compounds that are degraded by the bacteria in the GI tract and enhance the growth of endogenous beneficial bacteria, particularly lactobacilli and bifidobacteria. Generally, bacterial fermentation of prebiotics result in production of SCFAs that can be linked to their beneficial effects (Davani-Davari et al., 2019). Some examples of prebiotics are inulin, starch, pectin, galacto-oligosaccharides, and fructo-oligosaccharides. Although the use of prebiotics is well-established and health benefits are reported from their use, studies conducted with prebiotics in ASD patients are very few and the evidence provided is limited and non-conclusive (Grimaldi et al., 2017; Fattorusso et al., 2019).

### Fecal Microbiota Transplantation and Microbiota Transfer Therapy

Fecal microbiota transplantations (FMT) and MTT are two effective strategies for treating ASD symptoms. FMT is designed to alter the entire microbiome by transferring fecal material containing microbiota from a healthy donor to another person with an impaired gut microbiota. It has proved to be very successful in the treatment of recurrent *C. difficile* infections (CDI) (Kellingray et al., 2018) and is being developed for IBD and IBS treatments (Aroniadis and Brandt, 2013; Rossen et al., 2015) and other microbiota associated disorders. Therefore, it has attracted attention of researchers as potential treatment for children with ASD and currently FMT clinical trials are in progress. However, it requires careful development and consideration since some side effects are reported, including diarrhea, abdominal cramps, abdominal distress, and low fever (Kelly et al., 2015). Also, we cannot be certain about the long-term effect of FMT. The MTT is similar to FMT but comprises 14 days of antibiotic treatment and a process of bowel cleansing. There is also the administration of a standardized human gut microbiota (SHGM) for 7–8 weeks with an initial high dose. This technique has shown improvement of both GI and ASD-related symptoms, and normalized the microbiota of ASD patients (Kang et al., 2017).

### Dietary Interventions

One of the characteristics of children with ASD is the narrow diet, with a refusal of foods, based on its presentation or utensil use, and a limited food repertoire (Schreck and Williams, 2006; Bandini et al., 2010). The intake of fruits, vegetables, and proteins is less than in children with typical development and ASD children also ingest lower daily levels of potassium, copper, folate, and calcium when compared with controls (Sharp et al., 2013; Malhi et al., 2017). Diet is one of the most effective regulators of the gut microbiota and metabolite levels (Wu et al., 2011), and therefore, these behaviors are associated with lower levels of *Roseburia* spp. and *Eubacterium rectale*, linked to a lower intake of carbohydrates (Duncan et al., 2007; Wu et al., 2011; Tremaroli and Backhed, 2012). ASD patients who were treated with omega-3 FA for 12 weeks improved significantly their social behavior (Ooi et al., 2015). Another double-blind, placebo-controlled study showed that a treatment of levocarnitine for 3 months also improved ASD symptoms (Geier et al., 2011).

## Animal Models for the Study of the Relationship Between Gut Microbiota and ASD

Animal models can potentially play an important role in ASD research (Patel et al., 2018). There are many genetic models for the study of autistic disorders (Patel et al., 2018). However, there are no animal models that exhibit all the symptoms of human neurodevelopmental impairment. Experiments for the study of ASD have been conducted in zebrafish, monkeys, and songbirds, but mainly in rodents bred in laboratories, such as rats or mice (Hrabovska and Salyha, 2016). Rodents are suitable for the study of ASD because their behavior is well studied and there are a number of well-established techniques to manipulate their nervous system. Moreover, rats and mice are social animals and their relationships for parental, sexual or territorial behaviors, among others, are well-established. Initial ASD studies were conducted in rats, as their social behavior is clearly displayed. However, as mice are cheaper, their use for ASD study has been increasing. As a general rule, social behavior is measured by a series of tests such as the Morris water task, the three-chambered social interaction, swimming tests or simply by evaluating the explorative behaviors (Roullet and Crawley, 2011).

Despite the many ASD genetic models, the animal models used for the study of the gut microbiota-ASD relationship are more limited and are mostly inbred (e.g., BTBR) or environmental models (e.g., VPA, MIA) (Patel et al., 2018). Among the inbred mice model, BTBR mouse strain shows phenotypic traits of ASD symptoms and has been used extensively (Kratsman et al., 2016). It shows a consistent replication of ASD phenotype in different laboratories, and has been used in numerous studies assessing the effects of gut microbiota products and drugs on ASD-related outcomes (Kratsman et al., 2016). Another mice strain used for ASD studies is C57Bl/6J. However, this strain has been described as less impulsive and more motivated in comparison to BTBR (McTighe et al., 2013). Despite this, C57Bl/6J has been used in studies that have highlighted the alleviating effect of a probiotic strain on ASD behavior (Hsiao et al., 2013). During administration of *B. fragilis*, pregnant females of C57Bl/6J were also included as a maternal immune activation (MIA) mouse model. MIA during gestation has been shown to increase the risk of development of neurodevelopmental psychiatric disorders (Conway and Brown, 2019; Kreitz et al., 2020). This is of particular interest in ASD, as GI barrier impairment can lead to inflammatory processes that ultimately might affect the neurodevelopment of the offspring. Both BTBR and C57Bl/6J have been used simultaneously in different studies. One such study found that ketogenic diet modified the gut microbiota of BTBR mice, rebalancing the ratio of Firmicutes to Bacteroidetes and reduced *Akkermansia* levels (Newell et al., 2016). They were also used in a recent study that showed that such mice were able to develop autistic behavior after FMT using fecal material from human ASD (Sharon et al., 2019). BTBR mice were tested using four behavioral tests: open field testing, marble burying, three chamber sociability test, and ultrasonic vocalization, based on interactions in male-female context. The study showed that *Lachnospiraceae*, *Bacteroides*, and *Parabacteroides* were different between the ASD group and the typical development group. Moreover, the metabolite profiles were different between the two groups, especially in the case of 5-aminovaleric acid (5AV), a GABA receptor agonist produced by gut microbiota, that was significantly depleted. Administration of 5AV and taurine to BTBR mice restored excitability levels of pyramidal neurons, highlighting that these models can be used to monitor electrical alterations in the nervous system derived from the effect of the gut microbiota.

Long-Evans rats as ASD model were utilized for studies on PPA (Meeking et al., 2020). A study using Sprague Dawley rats, where VPA was delivered to pregnant rats to assess its effect on the gut microbial richness and diversity of the offspring, concluded that VPA induced microbiome traits for ASD and also the behavioral changes (Liu et al., 2018). Overall, the development of accurate models will be critical for the study of the gut microbiota traits in ASD.

## Concluding Remarks

The prevalence of ASD indicates that there is an urgent need to find new more effective treatments. Most of the research conducted so far has focused on alleviating ASD symptoms. The evidence suggesting a link between ASD and the gut microbiota, via the gut-brain axis, is now well-established. There are a number of pathways that are used in the microbiota-gut-brain axis connection. Understanding this connection opens the door to treatments and interventions that will improve the quality of life of patients and their families. It is likely that these interventions might not improve ASD-like symptoms when there are underlying genetic and environmental reasons, but they might help if the symptoms are gut microbiota-associated. At the moment, many clinical studies have shown that treatments for regulation of the gut microbiota provide improvements in ASD symptoms. However, biomarkers related to the gut microbiota activity have not been identified until recently. There is a need of more clinical and well-designed studies that include more patients, to provide more robust evidence that supports the use of probiotics, dietary and supplement treatments. In order to improve our understanding and design better studies, it is pivotal to identify robust gut microbiota-associated biomarkers.

## Author Contributions

EG-G, JR, and AN designed the manuscript. EG-G wrote the manuscript. JR and AN critically revised the manuscript and approved the final version. All authors contributed to the article and approved the submitted version.

## Conflict of Interest

The authors declare that the research was conducted in the absence of any commercial or financial relationships that could be construed as a potential conflict of interest.
